# Chlorido[2,3,5,6-tetra­kis­(*tert*-butyl­sulfanylmeth­yl)phenyl-κ^3^ *S* ^2^,*C* ^1^,*S* ^6^]palladium(II) dichloro­methane monosolvate

**DOI:** 10.1107/S1600536813003036

**Published:** 2013-02-06

**Authors:** Evelyn Paz-Morales, Simón Hernández-Ortega, David Morales-Morales

**Affiliations:** a Instituto de Química, Universidad Nacional Autónoma de México, Circuito Exterior, Ciudad Universitaria, Coyoacan, CP 04510, México, DF, Mexico

## Abstract

The title compound, [Pd(C_26_H_45_S_4_)Cl]·CH_2_Cl_2_, crystallizes with a disordered dichloro­methane solvent mol­ecule [occupancy ratio = 0.67 (4):0.33 (4)]. Two of the *tert*-butyl groups are also disordered [occupancy ratios = 0.70 (5):0.30 (5) and 0.63 (4):0.37 (4)]. Although the pincer ligand offers the possibility for coordination of two different metal atoms, the present structure shows only the coordination of a single Pd^II^ atom in a typical S—C—S tridentate pincer manner. The Pd^II^ atom is in a slightly distorted square-planar environment with the two *tert*-butyl­sulfanyl groups arranged in a *trans* con­formation and with a chloride ligand *trans* to the σ-bonded aromatic C atom. The structure exhibits a durene-like ligand frame, forming a dihedral angle of 13.6 (4)° with the metal coordination (Pd/S/S/Cl/C) environment. It is noteworthy that the *tert*-butyl groups are found in a *syn* arrangement, this being different to that found previously by Loeb, Shimizu & Wisner [(1998). *Organometallics*, **17**, 2324–2327].

## Related literature

For background to pincer compounds, see: Arroyo *et al.*, (2003 ▶); Errington, *et al.* (1980 ▶); Morales-Morales & Jensen (2007 ▶). For an isomeric structure, see: Loeb *et al.* (1998 ▶).
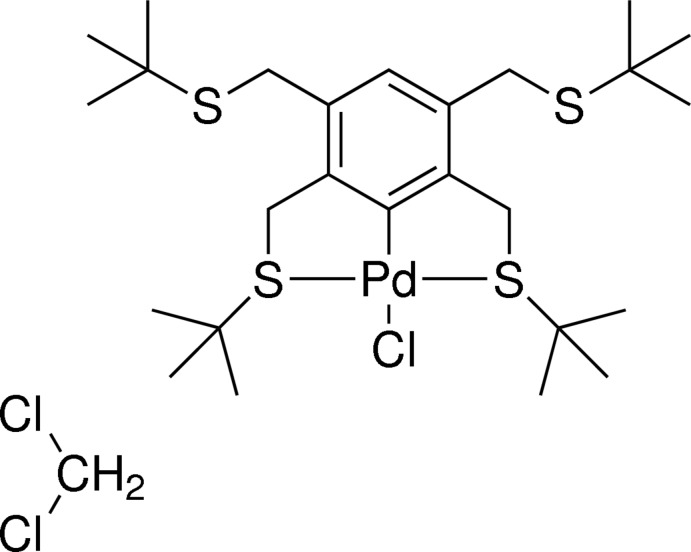


## Experimental

### 

#### Crystal data

[Pd(C_26_H_45_S_4_)Cl]·CH_2_Cl_2_
*M* *_r_* = 712.64
Monoclinic, 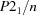

*a* = 15.917 (15) Å
*b* = 13.768 (13) Å
*c* = 17.808 (16) Å
β = 105.216 (15)°
*V* = 3766 (6) Å^3^
*Z* = 4
Mo *K*α radiation
μ = 0.94 mm^−1^
*T* = 298 K
0.27 × 0.22 × 0.15 mm

#### Data collection

Bruker SMART APEX CCD diffractometer
Absorption correction: integration (*SADABS*; Sheldrick, 1996 ▶) *T*_min_ = 0.593, *T*_max_ = 0.745
28207 measured reflections
6570 independent reflections
5144 reflections with *I* > 2σ(*I*)
*R*_int_ = 0.066

#### Refinement

*R*[*F* ^2^ > 2σ(*F* ^2^)] = 0.048
*wR*(*F* ^2^) = 0.110
*S* = 1.03
6570 reflections
406 parameters
291 restraints
H-atom parameters constrained
Δρ_max_ = 0.64 e Å^−3^
Δρ_min_ = −0.24 e Å^−3^

### 

Data collection: *SMART* (Bruker, 1999 ▶); cell refinement: *SAINT-Plus* (Bruker, 1999 ▶); data reduction: *SAINT-Plus*; program(s) used to solve structure: *SHELXS97* (Sheldrick, 2008 ▶); program(s) used to refine structure: *SHELXL97* (Sheldrick, 2008 ▶); molecular graphics: *ORTEP-3 for Windows* (Farrugia, 2012 ▶); software used to prepare material for publication: *SHELXTL* (Sheldrick, 2008 ▶).

## Supplementary Material

Crystal structure: contains datablock(s) I, global. DOI: 10.1107/S1600536813003036/pk2463sup1.cif

Structure factors: contains datablock(s) I. DOI: 10.1107/S1600536813003036/pk2463Isup2.hkl

Additional supplementary materials:  crystallographic information; 3D view; checkCIF report

## Figures and Tables

**Table 1:** Selected bond lengths (Å)

Pd—C2	2.022 (4)
Pd—S1	2.326 (2)
Pd—S2	2.333 (2)
Pd—Cl1	2.441 (2)

**Fig. 1.**
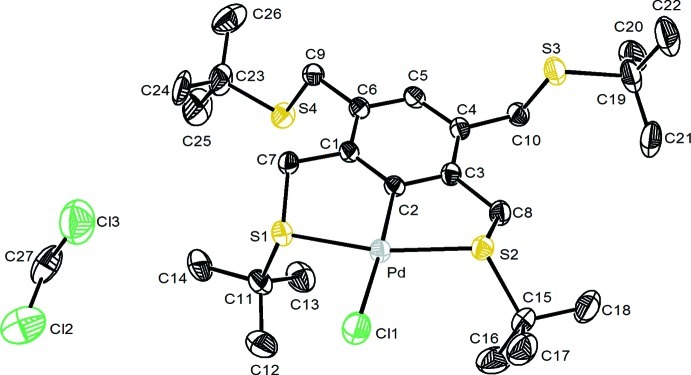
The molecular structure of (I). Displacement ellipsoids are drawn at the 40% probability level. The H-atoms and the minor disorder components have been omitted to enhance clarity.

## supplementary crystallographic information

### Comment

Pincer compounds have had a preponderant importance in chemistry, this being especially important in areas such as homogeneous catalysis, organometallic chemistry, the activation of unreactive or difficult to activate bonds and the activation of small molecules (Errington *et al.* 1980; Morales-Morales & Jensen, 2007). Among these species, those including sulfur as donor atom have been scarcely studied (Arroyo *et al.*, 2003), mostly due to the well known tendency of sulfur to kill the activity of homogeneous catalysts. Thus, following our continuing interest in the synthesis of pincer compounds, we report here the crystal structure of the Pd(II) sulfur based pincer complex (1,2,4,5-tetrakis(*tert*-butylsulfanylmethyl)phenyl)-chloro-palladium (II) dichloromethane solvate (I).

The structure of (I) is shown in Fig. 1 with the numbering scheme. Compound (I) was crystallized as a dichloromethane solvate. Selected bond distances and angles are shown in Table 1. In agreement with the dihedral angles of the planes C7—C1—C2—C3—C8 and S1—Pd—S2—Cl1 (13.6 (4)°) the Pd atom is located in a slightly distorted square-planar geometry. Compound (I) is a geometric isomer of a previously described compound (Loeb *et al.* 1998). However, there are several noticeable differences in our compound (I), the *tert*-butyl substituents at the S are found in a *syn* fashion while those described in the previously reported compound are oriented in an *anti* fashion with respect to the square plane. The bond distances for Pd—S and Pd—C [2.326 (2) Å, 2.333 (2) Å and 2.022 (4) Å] are slightly larger than those of the *anti* isomer [2.297 (3) Å, 2.302 (3) Å and 1.994 (4) Å]. The Pd—Cl bond distance [2.441 (2) Å] is larger than of the *anti* isomer. While the uncoordinated *tert*-butylS groups are found in a similar geometry as those previously observed in the Loeb's species (Loeb *et al.* 1998). Finally, both the *tert*-butyl groups and the dichloromethane solvent molecule are disordered and were refined as disordered with two components.

### Experimental

The title compound was synthesized according to a published procedure (Loeb *et al.* 1998). Crystals suitable for X-ray diffraction analysis were obtained by slow evaporation of a solution of the title compound (I) in CH_2_Cl_2_.

### Refinement

Two *tert*-butyl and CH_2_Cl_2_ solvent, are disordered and were modelled and refined in two major contributors. The ratio of S.O.F., were 70/30 and 63/37 for tertbutyl groups and 67/33 for CH_2_Cl_2_ solvent. H atoms on C atoms were included in calculated positions (C—H = 0.93 Å for C—H arom., 0.97 Å for CH_2_, and 0.96 Å for CH_3_), H atoms were refined using a riding model, with *U*_iso_(H) = 1.2*U*_eq_ of the carrier atom for C—H-arom. and methylene groups and *U*_iso_(H) = 1.5*U*_eq_ for methyl groups.

### Crystal data

**Table:**

[Pd(C_26_H_45_S_4_)Cl]·CH_2_Cl_2_	*F*(000) = 1480
*M**_r_* = 712.64	*D*_x_ = 1.257 Mg m^−^^3^
Monoclinic, *P*2_1_/*n*	Mo *K*α radiation, λ = 0.71073 Å
Hall symbol: -P 2yn	Cell parameters from 8648 reflections
*a* = 15.917 (15) Å	θ = 2.4–24.8°
*b* = 13.768 (13) Å	µ = 0.94 mm^−^^1^
*c* = 17.808 (16) Å	*T* = 298 K
β = 105.216 (15)°	Prism, colourless
*V* = 3766 (6) Å^3^	0.27 × 0.22 × 0.15 mm
*Z* = 4	

### Data collection

**Table:**

Bruker SMART APEX CCD diffractometer	6570 independent reflections
Radiation source: fine-focus sealed tube	5144 reflections with *I* > 2σ(*I*)
Graphite monochromator	*R*_int_ = 0.066
Detector resolution: 0.83 pixels mm^-1^	θ_max_ = 25.0°, θ_min_ = 2.0°
ω scans	*h* = −18→18
Absorption correction: integration (*SADABS*; Sheldrick, 1996)	*k* = −16→16
*T*_min_ = 0.593, *T*_max_ = 0.745	*l* = −20→20
28207 measured reflections	

### Refinement

**Table:**

Refinement on *F*^2^	Primary atom site location: structure-invariant direct methods
Least-squares matrix: full	Secondary atom site location: difference Fourier map
*R*[*F*^2^ > 2σ(*F*^2^)] = 0.048	Hydrogen site location: inferred from neighbouring sites
*wR*(*F*^2^) = 0.110	H-atom parameters constrained
*S* = 1.03	*w* = 1/[σ^2^(*F*_o_^2^) + (0.055*P*)^2^] where *P* = (*F*_o_^2^ + 2*F*_c_^2^)/3
6570 reflections	(Δ/σ)_max_ = 0.002
406 parameters	Δρ_max_ = 0.64 e Å^−^^3^
291 restraints	Δρ_min_ = −0.24 e Å^−^^3^

### Special details

**Table:**

Geometry. All e.s.d.'s (except the e.s.d. in the dihedral angle between two l.s. planes) are estimated using the full covariance matrix. The cell e.s.d.'s are taken into account individually in the estimation of e.s.d.'s in distances, angles and torsion angles; correlations between e.s.d.'s in cell parameters are only used when they are defined by crystal symmetry. An approximate (isotropic) treatment of cell e.s.d.'s is used for estimating e.s.d.'s involving l.s. planes.
Refinement. Refinement of *F*^2^ against ALL reflections. The weighted *R*-factor *wR* and goodness of fit *S* are based on *F*^2^, conventional *R*-factors *R* are based on *F*, with *F* set to zero for negative *F*^2^. The threshold expression of *F*^2^ > σ(*F*^2^) is used only for calculating *R*-factors(gt) *etc*. and is not relevant to the choice of reflections for refinement. *R*-factors based on *F*^2^ are statistically about twice as large as those based on *F*, and *R*- factors based on ALL data will be even larger.

### Fractional atomic coordinates and isotropic or equivalent isotropic displacement parameters (Å^2^)

**Table:**

	*x*	*y*	*z*	*U*_iso_*/*U*_eq_	Occ. (<1)
Pd	0.386615 (17)	1.08106 (2)	0.319516 (16)	0.04396 (12)	
Cl1	0.37582 (7)	1.23743 (9)	0.25300 (8)	0.0792 (4)	
S1	0.53435 (6)	1.06823 (7)	0.32714 (6)	0.0491 (2)	
S2	0.24590 (6)	1.07447 (7)	0.33560 (5)	0.0476 (2)	
S3	0.23346 (7)	0.83956 (10)	0.53990 (7)	0.0700 (3)	
S4	0.62344 (7)	0.72277 (8)	0.41972 (6)	0.0623 (3)	
C1	0.4886 (2)	0.9199 (3)	0.41609 (19)	0.0418 (8)	
C2	0.4033 (2)	0.9564 (3)	0.38174 (19)	0.0393 (8)	
C3	0.3296 (2)	0.9071 (3)	0.3951 (2)	0.0428 (9)	
C4	0.3422 (2)	0.8228 (3)	0.4436 (2)	0.0437 (9)	
C5	0.4270 (2)	0.7883 (3)	0.4761 (2)	0.0466 (9)	
H5	0.4347	0.7332	0.5073	0.056*	
C6	0.5011 (2)	0.8349 (3)	0.46264 (19)	0.0440 (9)	
C7	0.5669 (2)	0.9770 (3)	0.4048 (2)	0.0469 (9)	
H7A	0.6081	0.9323	0.3920	0.056*	
H7B	0.5957	1.0092	0.4532	0.056*	
C8	0.2386 (2)	0.9439 (3)	0.3549 (2)	0.0522 (10)	
H8A	0.1997	0.9333	0.3880	0.063*	
H8B	0.2157	0.9092	0.3064	0.063*	
C9	0.5909 (2)	0.7917 (3)	0.4969 (2)	0.0492 (9)	
H9A	0.5896	0.7488	0.5398	0.059*	
H9B	0.6326	0.8430	0.5165	0.059*	
C10	0.2641 (2)	0.7715 (3)	0.4616 (2)	0.0532 (10)	
H10A	0.2795	0.7053	0.4781	0.064*	
H10B	0.2156	0.7698	0.4154	0.064*	
C11	0.5483 (3)	1.0033 (3)	0.2379 (2)	0.0626 (11)	
C12	0.5103 (4)	1.0728 (4)	0.1686 (3)	0.0945 (17)	
H12A	0.5158	1.0434	0.1212	0.142*	
H12B	0.5418	1.1330	0.1768	0.142*	
H12C	0.4500	1.0848	0.1650	0.142*	
C13	0.4989 (3)	0.9058 (4)	0.2249 (3)	0.0851 (16)	
H13A	0.4376	0.9176	0.2159	0.128*	
H13B	0.5188	0.8656	0.2702	0.128*	
H13C	0.5096	0.8735	0.1805	0.128*	
C14	0.6471 (3)	0.9881 (4)	0.2493 (3)	0.0868 (16)	
H14A	0.6683	0.9415	0.2900	0.130*	
H14B	0.6769	1.0487	0.2632	0.130*	
H14C	0.6574	0.9646	0.2017	0.130*	
C15	0.1526 (2)	1.0958 (3)	0.2465 (2)	0.0613 (11)	
C16	0.1716 (3)	1.0506 (4)	0.1738 (3)	0.0981 (18)	
H16A	0.1257	1.0671	0.1288	0.147*	
H16B	0.1751	0.9812	0.1795	0.147*	
H16C	0.2259	1.0752	0.1678	0.147*	
C17	0.1440 (3)	1.2083 (4)	0.2396 (3)	0.0865 (15)	
H17A	0.1006	1.2249	0.1929	0.130*	
H17B	0.1989	1.2358	0.2380	0.130*	
H17C	0.1272	1.2335	0.2838	0.130*	
C18	0.0705 (3)	1.0526 (4)	0.2631 (3)	0.0928 (17)	
H18A	0.0656	1.0749	0.3128	0.139*	
H18B	0.0742	0.9830	0.2634	0.139*	
H18C	0.0202	1.0729	0.2233	0.139*	
C19	0.1205 (3)	0.7963 (4)	0.5345 (3)	0.1000 (16)	
C20	0.1243 (12)	0.6833 (5)	0.5442 (11)	0.113 (3)	0.70 (5)
H20A	0.1444	0.6548	0.5029	0.170*	0.70 (5)
H20B	0.0672	0.6591	0.5423	0.170*	0.70 (5)
H20C	0.1635	0.6668	0.5934	0.170*	0.70 (5)
C21	0.0584 (8)	0.8249 (16)	0.4531 (7)	0.112 (4)	0.70 (5)
H21A	0.0764	0.7916	0.4126	0.167*	0.70 (5)
H21B	0.0613	0.8938	0.4457	0.167*	0.70 (5)
H21C	−0.0003	0.8069	0.4515	0.167*	0.70 (5)
C22	0.0923 (15)	0.8477 (12)	0.6022 (10)	0.142 (4)	0.70 (5)
H22A	0.0890	0.9165	0.5933	0.213*	0.70 (5)
H22B	0.1343	0.8343	0.6506	0.213*	0.70 (5)
H22C	0.0363	0.8238	0.6044	0.213*	0.70 (5)
C20A	0.101 (3)	0.6851 (9)	0.522 (2)	0.119 (6)	0.30 (5)
H20D	0.1409	0.6493	0.5629	0.179*	0.30 (5)
H20E	0.1090	0.6656	0.4728	0.179*	0.30 (5)
H20F	0.0426	0.6721	0.5239	0.179*	0.30 (5)
C21A	0.0545 (19)	0.858 (3)	0.473 (2)	0.120 (6)	0.30 (5)
H21D	0.0563	0.8385	0.4213	0.180*	0.30 (5)
H21E	0.0698	0.9257	0.4798	0.180*	0.30 (5)
H21F	−0.0032	0.8488	0.4784	0.180*	0.30 (5)
C22A	0.122 (3)	0.828 (3)	0.6194 (11)	0.125 (6)	0.30 (5)
H22D	0.1622	0.7884	0.6561	0.188*	0.30 (5)
H22E	0.0645	0.8204	0.6270	0.188*	0.30 (5)
H22F	0.1390	0.8949	0.6270	0.188*	0.30 (5)
C23	0.7421 (3)	0.7020 (3)	0.4609 (3)	0.0885 (14)	
C24	0.7840 (11)	0.8059 (7)	0.4636 (12)	0.097 (3)	0.63 (4)
H24A	0.7718	0.8320	0.4119	0.145*	0.63 (4)
H24B	0.8459	0.8012	0.4850	0.145*	0.63 (4)
H24C	0.7600	0.8479	0.4957	0.145*	0.63 (4)
C25	0.7747 (14)	0.6346 (11)	0.4031 (11)	0.120 (4)	0.63 (4)
H25A	0.7607	0.6638	0.3524	0.180*	0.63 (4)
H25B	0.7468	0.5724	0.4002	0.180*	0.63 (4)
H25C	0.8367	0.6264	0.4214	0.180*	0.63 (4)
C26	0.7646 (13)	0.6563 (17)	0.5444 (7)	0.118 (4)	0.63 (4)
H26A	0.7395	0.5925	0.5418	0.177*	0.63 (4)
H26B	0.7413	0.6964	0.5781	0.177*	0.63 (4)
H26C	0.8266	0.6518	0.5643	0.177*	0.63 (4)
C24A	0.8068 (18)	0.7877 (18)	0.4898 (18)	0.112 (5)	0.37 (4)
H24D	0.8018	0.8339	0.4485	0.169*	0.37 (4)
H24E	0.8652	0.7631	0.5054	0.169*	0.37 (4)
H24F	0.7930	0.8189	0.5334	0.169*	0.37 (4)
C25A	0.759 (2)	0.653 (2)	0.3868 (13)	0.108 (5)	0.37 (4)
H25D	0.7200	0.5991	0.3711	0.162*	0.37 (4)
H25E	0.8183	0.6293	0.3988	0.162*	0.37 (4)
H25F	0.7504	0.6991	0.3454	0.162*	0.37 (4)
C26A	0.747 (2)	0.6253 (19)	0.5270 (14)	0.108 (5)	0.37 (4)
H26D	0.7079	0.5727	0.5073	0.162*	0.37 (4)
H26E	0.7310	0.6554	0.5698	0.162*	0.37 (4)
H26F	0.8054	0.6007	0.5444	0.162*	0.37 (4)
Cl2	0.8993 (11)	0.9229 (8)	0.1142 (5)	0.148 (3)	0.67 (4)
Cl3	0.8721 (9)	0.8710 (11)	0.2647 (7)	0.176 (3)	0.67 (4)
C27	0.9079 (19)	0.8314 (12)	0.1845 (13)	0.113 (5)	0.67 (4)
H27A	0.9682	0.8108	0.2023	0.135*	0.67 (4)
H27B	0.8736	0.7758	0.1610	0.135*	0.67 (4)
Cl2A	0.875 (2)	0.9294 (16)	0.1197 (14)	0.172 (7)	0.33 (4)
Cl3A	0.841 (2)	0.835 (3)	0.2547 (12)	0.185 (6)	0.33 (4)
C27A	0.890 (4)	0.824 (2)	0.177 (2)	0.104 (6)	0.33 (4)
H27C	0.9513	0.8112	0.1972	0.125*	0.33 (4)
H27D	0.8640	0.7690	0.1446	0.125*	0.33 (4)

### Atomic displacement parameters (Å^2^)

**Table:**

	*U*^11^	*U*^22^	*U*^33^	*U*^12^	*U*^13^	*U*^23^
Pd	0.04006 (17)	0.0427 (2)	0.04995 (18)	−0.00192 (13)	0.01323 (13)	0.00124 (14)
Cl1	0.0618 (7)	0.0613 (8)	0.1110 (9)	0.0010 (6)	0.0164 (6)	0.0313 (7)
S1	0.0453 (5)	0.0476 (6)	0.0576 (6)	−0.0058 (4)	0.0191 (5)	0.0012 (5)
S2	0.0428 (5)	0.0499 (6)	0.0497 (5)	0.0032 (4)	0.0116 (4)	−0.0025 (5)
S3	0.0543 (6)	0.0934 (10)	0.0689 (7)	−0.0112 (6)	0.0282 (6)	−0.0068 (6)
S4	0.0554 (6)	0.0693 (8)	0.0635 (6)	0.0091 (6)	0.0181 (5)	−0.0143 (6)
C1	0.0394 (19)	0.051 (2)	0.0380 (18)	−0.0016 (18)	0.0147 (15)	−0.0026 (17)
C2	0.042 (2)	0.041 (2)	0.0371 (18)	0.0014 (17)	0.0144 (15)	−0.0010 (16)
C3	0.0387 (19)	0.045 (2)	0.047 (2)	0.0026 (17)	0.0145 (16)	−0.0026 (17)
C4	0.041 (2)	0.047 (2)	0.046 (2)	−0.0024 (17)	0.0163 (16)	−0.0029 (18)
C5	0.051 (2)	0.048 (2)	0.046 (2)	0.0025 (19)	0.0205 (18)	0.0046 (18)
C6	0.043 (2)	0.051 (2)	0.0412 (19)	0.0037 (18)	0.0162 (16)	−0.0054 (17)
C7	0.0375 (19)	0.058 (3)	0.046 (2)	0.0004 (18)	0.0125 (16)	−0.0029 (18)
C8	0.043 (2)	0.052 (3)	0.063 (2)	−0.0019 (18)	0.0161 (19)	0.008 (2)
C9	0.046 (2)	0.056 (3)	0.047 (2)	0.0067 (19)	0.0146 (17)	0.0025 (18)
C10	0.051 (2)	0.053 (3)	0.058 (2)	−0.0036 (19)	0.0172 (19)	0.010 (2)
C11	0.065 (3)	0.076 (3)	0.055 (2)	−0.004 (2)	0.029 (2)	0.002 (2)
C12	0.100 (4)	0.123 (5)	0.068 (3)	0.011 (3)	0.037 (3)	0.017 (3)
C13	0.099 (4)	0.094 (4)	0.072 (3)	−0.018 (3)	0.039 (3)	−0.024 (3)
C14	0.072 (3)	0.116 (4)	0.090 (4)	0.008 (3)	0.052 (3)	0.002 (3)
C15	0.040 (2)	0.075 (3)	0.063 (3)	0.009 (2)	0.0042 (19)	0.009 (2)
C16	0.102 (4)	0.129 (5)	0.054 (3)	0.018 (4)	0.005 (3)	−0.009 (3)
C17	0.066 (3)	0.084 (4)	0.104 (4)	0.019 (3)	0.011 (3)	0.020 (3)
C18	0.046 (3)	0.112 (5)	0.111 (4)	0.000 (3)	0.003 (3)	0.020 (3)
C19	0.062 (3)	0.157 (4)	0.096 (3)	−0.022 (3)	0.047 (2)	−0.006 (3)
C20	0.091 (8)	0.157 (5)	0.096 (7)	−0.070 (5)	0.033 (6)	0.008 (4)
C21	0.045 (4)	0.178 (9)	0.117 (5)	0.006 (5)	0.030 (4)	−0.012 (6)
C22	0.083 (9)	0.241 (9)	0.125 (6)	−0.008 (7)	0.067 (6)	−0.030 (7)
C20A	0.094 (13)	0.165 (6)	0.088 (11)	−0.055 (9)	0.005 (11)	0.011 (8)
C21A	0.069 (10)	0.176 (11)	0.128 (9)	0.008 (10)	0.047 (9)	−0.006 (10)
C22A	0.070 (13)	0.215 (13)	0.114 (6)	−0.018 (10)	0.065 (8)	−0.024 (8)
C23	0.051 (3)	0.121 (4)	0.100 (3)	0.018 (2)	0.030 (3)	−0.023 (3)
C24	0.028 (6)	0.155 (5)	0.107 (8)	−0.014 (5)	0.017 (6)	−0.038 (5)
C25	0.081 (8)	0.147 (7)	0.144 (7)	0.045 (7)	0.050 (7)	−0.033 (7)
C26	0.087 (8)	0.144 (9)	0.103 (5)	0.041 (7)	−0.011 (6)	−0.008 (6)
C24A	0.043 (10)	0.168 (9)	0.115 (12)	−0.001 (7)	0.002 (9)	−0.038 (8)
C25A	0.073 (10)	0.135 (11)	0.125 (8)	0.041 (9)	0.043 (9)	−0.036 (7)
C26A	0.082 (11)	0.129 (10)	0.089 (8)	0.042 (8)	−0.019 (8)	−0.013 (7)
Cl2	0.184 (7)	0.102 (3)	0.134 (3)	0.001 (4)	0.001 (4)	−0.036 (3)
Cl3	0.126 (5)	0.218 (7)	0.196 (4)	−0.003 (5)	0.060 (4)	−0.015 (4)
C27	0.069 (12)	0.082 (5)	0.165 (6)	0.015 (6)	−0.008 (7)	−0.026 (5)
Cl2A	0.190 (14)	0.102 (7)	0.178 (10)	0.041 (7)	−0.035 (7)	0.010 (7)
Cl3A	0.122 (11)	0.254 (16)	0.191 (7)	0.042 (9)	0.062 (8)	−0.039 (9)
C27A	0.072 (16)	0.085 (8)	0.145 (8)	0.012 (9)	0.011 (10)	−0.018 (7)

### Geometric parameters (Å, º)

**Table:**

Pd—C2	2.022 (4)	C19—C21A	1.563 (7)
Pd—S1	2.326 (2)	C19—C20	1.564 (6)
Pd—S2	2.333 (2)	C19—C20A	1.565 (8)
Pd—Cl1	2.441 (2)	C19—C22A	1.570 (7)
S1—C7	1.839 (4)	C19—C21	1.574 (6)
S1—C11	1.887 (4)	C20—H20A	0.9600
S2—C8	1.839 (4)	C20—H20B	0.9600
S2—C15	1.890 (4)	C20—H20C	0.9600
S3—C10	1.849 (4)	C21—H21A	0.9600
S3—C19	1.873 (5)	C21—H21B	0.9600
S4—C9	1.852 (4)	C21—H21C	0.9600
S4—C23	1.861 (5)	C22—H22A	0.9600
C1—C6	1.418 (5)	C22—H22B	0.9600
C1—C2	1.427 (5)	C22—H22C	0.9600
C1—C7	1.530 (5)	C20A—H20D	0.9600
C2—C3	1.428 (5)	C20A—H20E	0.9600
C3—C4	1.429 (5)	C20A—H20F	0.9600
C3—C8	1.525 (5)	C21A—H21D	0.9600
C4—C5	1.405 (5)	C21A—H21E	0.9600
C4—C10	1.534 (5)	C21A—H21F	0.9600
C5—C6	1.418 (5)	C22A—H22D	0.9600
C5—H5	0.9300	C22A—H22E	0.9600
C6—C9	1.521 (5)	C22A—H22F	0.9600
C7—H7A	0.9700	C23—C24A	1.561 (7)
C7—H7B	0.9700	C23—C26	1.566 (7)
C8—H8A	0.9700	C23—C26A	1.567 (7)
C8—H8B	0.9700	C23—C25A	1.572 (7)
C9—H9A	0.9700	C23—C25	1.572 (6)
C9—H9B	0.9700	C23—C24	1.574 (6)
C10—H10A	0.9700	C24—H24A	0.9600
C10—H10B	0.9700	C24—H24B	0.9600
C11—C13	1.542 (6)	C24—H24C	0.9600
C11—C14	1.546 (6)	C25—H25A	0.9600
C11—C12	1.553 (6)	C25—H25B	0.9600
C12—H12A	0.9600	C25—H25C	0.9600
C12—H12B	0.9600	C26—H26A	0.9600
C12—H12C	0.9600	C26—H26B	0.9600
C13—H13A	0.9600	C26—H26C	0.9600
C13—H13B	0.9600	C24A—H24D	0.9600
C13—H13C	0.9600	C24A—H24E	0.9600
C14—H14A	0.9600	C24A—H24F	0.9600
C14—H14B	0.9600	C25A—H25D	0.9600
C14—H14C	0.9600	C25A—H25E	0.9600
C15—C18	1.534 (6)	C25A—H25F	0.9600
C15—C16	1.536 (6)	C26A—H26D	0.9600
C15—C17	1.557 (6)	C26A—H26E	0.9600
C16—H16A	0.9600	C26A—H26F	0.9600
C16—H16B	0.9600	Cl2—C27	1.755 (8)
C16—H16C	0.9600	Cl3—C27	1.759 (8)
C17—H17A	0.9600	C27—H27A	0.9700
C17—H17B	0.9600	C27—H27B	0.9700
C17—H17C	0.9600	Cl2A—C27A	1.755 (10)
C18—H18A	0.9600	Cl3A—C27A	1.757 (10)
C18—H18B	0.9600	C27A—H27C	0.9700
C18—H18C	0.9600	C27A—H27D	0.9700
C19—C22	1.562 (6)		
			
C2—Pd—S1	85.33 (10)	C22—C19—C21	111.0 (5)
C2—Pd—S2	83.78 (10)	C20—C19—C21	110.4 (5)
S1—Pd—S2	168.00 (4)	C20A—C19—C21	93.8 (13)
C2—Pd—Cl1	175.31 (10)	C22A—C19—C21	131.5 (13)
S1—Pd—Cl1	92.10 (4)	C22—C19—S3	106.4 (8)
S2—Pd—Cl1	98.41 (4)	C21A—C19—S3	108.7 (14)
C7—S1—C11	103.7 (2)	C20—C19—S3	107.6 (7)
C7—S1—Pd	100.17 (11)	C20A—C19—S3	117.9 (17)
C11—S1—Pd	109.11 (14)	C22A—C19—S3	95.3 (13)
C8—S2—C15	103.53 (19)	C21—C19—S3	109.3 (6)
C8—S2—Pd	99.85 (12)	C19—C20—H20A	109.5
C15—S2—Pd	117.30 (14)	C19—C20—H20B	109.5
C10—S3—C19	103.9 (2)	H20A—C20—H20B	109.5
C9—S4—C23	103.79 (18)	C19—C20—H20C	109.5
C6—C1—C2	120.9 (3)	H20A—C20—H20C	109.5
C6—C1—C7	120.4 (3)	H20B—C20—H20C	109.5
C2—C1—C7	118.6 (3)	C19—C21—H21A	109.5
C1—C2—C3	119.5 (3)	C19—C21—H21B	109.5
C1—C2—Pd	120.4 (3)	H21A—C21—H21B	109.5
C3—C2—Pd	120.0 (3)	C19—C21—H21C	109.5
C2—C3—C4	119.7 (3)	H21A—C21—H21C	109.5
C2—C3—C8	119.0 (3)	H21B—C21—H21C	109.5
C4—C3—C8	121.3 (3)	C19—C22—H22A	109.5
C5—C4—C3	119.4 (3)	C19—C22—H22B	109.5
C5—C4—C10	120.1 (3)	H22A—C22—H22B	109.5
C3—C4—C10	120.5 (3)	C19—C22—H22C	109.5
C4—C5—C6	122.0 (3)	H22A—C22—H22C	109.5
C4—C5—H5	119.0	H22B—C22—H22C	109.5
C6—C5—H5	119.0	C19—C20A—H20D	109.5
C1—C6—C5	118.5 (3)	C19—C20A—H20E	109.5
C1—C6—C9	122.1 (3)	H20D—C20A—H20E	109.5
C5—C6—C9	119.4 (3)	C19—C20A—H20F	109.5
C1—C7—S1	111.8 (2)	H20D—C20A—H20F	109.5
C1—C7—H7A	109.3	H20E—C20A—H20F	109.5
S1—C7—H7A	109.3	C19—C21A—H21D	109.5
C1—C7—H7B	109.3	C19—C21A—H21E	109.5
S1—C7—H7B	109.3	H21D—C21A—H21E	109.5
H7A—C7—H7B	107.9	C19—C21A—H21F	109.5
C3—C8—S2	108.3 (3)	H21D—C21A—H21F	109.5
C3—C8—H8A	110.0	H21E—C21A—H21F	109.5
S2—C8—H8A	110.0	C19—C22A—H22D	109.5
C3—C8—H8B	110.0	C19—C22A—H22E	109.5
S2—C8—H8B	110.0	H22D—C22A—H22E	109.5
H8A—C8—H8B	108.4	C19—C22A—H22F	109.5
C6—C9—S4	108.6 (2)	H22D—C22A—H22F	109.5
C6—C9—H9A	110.0	H22E—C22A—H22F	109.5
S4—C9—H9A	110.0	C24A—C23—C26	91.4 (10)
C6—C9—H9B	110.0	C24A—C23—C26A	111.3 (6)
S4—C9—H9B	110.0	C24A—C23—C25A	111.0 (6)
H9A—C9—H9B	108.4	C26—C23—C25A	125.2 (15)
C4—C10—S3	108.2 (3)	C26A—C23—C25A	110.7 (6)
C4—C10—H10A	110.1	C24A—C23—C25	111.1 (16)
S3—C10—H10A	110.1	C26—C23—C25	110.6 (5)
C4—C10—H10B	110.1	C26A—C23—C25	97.7 (14)
S3—C10—H10B	110.1	C26—C23—C24	110.3 (5)
H10A—C10—H10B	108.4	C26A—C23—C24	130.7 (10)
C13—C11—C14	111.3 (4)	C25A—C23—C24	104.7 (14)
C13—C11—C12	109.9 (4)	C25—C23—C24	110.3 (5)
C14—C11—C12	111.0 (4)	C24A—C23—S4	121.8 (14)
C13—C11—S1	111.8 (3)	C26—C23—S4	113.0 (8)
C14—C11—S1	107.1 (3)	C26A—C23—S4	104.2 (12)
C12—C11—S1	105.7 (3)	C25A—C23—S4	96.8 (13)
C11—C12—H12A	109.5	C25—C23—S4	107.9 (9)
C11—C12—H12B	109.5	C24—C23—S4	104.5 (8)
H12A—C12—H12B	109.5	C23—C24—H24A	109.5
C11—C12—H12C	109.5	C23—C24—H24B	109.5
H12A—C12—H12C	109.5	H24A—C24—H24B	109.5
H12B—C12—H12C	109.5	C23—C24—H24C	109.5
C11—C13—H13A	109.5	H24A—C24—H24C	109.5
C11—C13—H13B	109.5	H24B—C24—H24C	109.5
H13A—C13—H13B	109.5	C23—C25—H25A	109.5
C11—C13—H13C	109.5	C23—C25—H25B	109.5
H13A—C13—H13C	109.5	H25A—C25—H25B	109.5
H13B—C13—H13C	109.5	C23—C25—H25C	109.5
C11—C14—H14A	109.5	H25A—C25—H25C	109.5
C11—C14—H14B	109.5	H25B—C25—H25C	109.5
H14A—C14—H14B	109.5	C23—C26—H26A	109.5
C11—C14—H14C	109.5	C23—C26—H26B	109.5
H14A—C14—H14C	109.5	H26A—C26—H26B	109.5
H14B—C14—H14C	109.5	C23—C26—H26C	109.5
C18—C15—C16	111.8 (4)	H26A—C26—H26C	109.5
C18—C15—C17	109.8 (4)	H26B—C26—H26C	109.5
C16—C15—C17	111.7 (4)	C23—C24A—H24D	109.5
C18—C15—S2	107.4 (3)	C23—C24A—H24E	109.5
C16—C15—S2	111.1 (3)	H24D—C24A—H24E	109.5
C17—C15—S2	104.7 (3)	C23—C24A—H24F	109.5
C15—C16—H16A	109.5	H24D—C24A—H24F	109.5
C15—C16—H16B	109.5	H24E—C24A—H24F	109.5
H16A—C16—H16B	109.5	C23—C25A—H25D	109.5
C15—C16—H16C	109.5	C23—C25A—H25E	109.5
H16A—C16—H16C	109.5	H25D—C25A—H25E	109.5
H16B—C16—H16C	109.5	C23—C25A—H25F	109.5
C15—C17—H17A	109.5	H25D—C25A—H25F	109.5
C15—C17—H17B	109.5	H25E—C25A—H25F	109.5
H17A—C17—H17B	109.5	C23—C26A—H26D	109.5
C15—C17—H17C	109.5	C23—C26A—H26E	109.5
H17A—C17—H17C	109.5	H26D—C26A—H26E	109.5
H17B—C17—H17C	109.5	C23—C26A—H26F	109.5
C15—C18—H18A	109.5	H26D—C26A—H26F	109.5
C15—C18—H18B	109.5	H26E—C26A—H26F	109.5
H18A—C18—H18B	109.5	Cl2—C27—Cl3	112.1 (5)
C15—C18—H18C	109.5	Cl2—C27—H27A	109.2
H18A—C18—H18C	109.5	Cl3—C27—H27A	109.2
H18B—C18—H18C	109.5	Cl2—C27—H27B	109.2
C22—C19—C21A	91.2 (13)	Cl3—C27—H27B	109.2
C22—C19—C20	112.0 (5)	H27A—C27—H27B	107.9
C21A—C19—C20	128.4 (15)	Cl2A—C27A—Cl3A	111.7 (7)
C22—C19—C20A	117.7 (19)	Cl2A—C27A—H27C	109.3
C21A—C19—C20A	111.5 (7)	Cl3A—C27A—H27C	109.3
C21A—C19—C22A	111.3 (7)	Cl2A—C27A—H27D	109.3
C20—C19—C22A	100.4 (16)	Cl3A—C27A—H27D	109.3
C20A—C19—C22A	111.0 (7)	H27C—C27A—H27D	107.9
